# Oral Polymorphonuclear Neutrophil Contributes to Oral Health

**DOI:** 10.1007/s40496-018-0199-6

**Published:** 2018-10-25

**Authors:** Patrick Rijkschroeff, Bruno G. Loos, Elena A. Nicu

**Affiliations:** 1 Department of Periodontology, Academic Centre for Dentistry Amsterdam (ACTA), University of Amsterdam and VU University Amsterdam, Gustav Mahlerlaan 3004, 1081 LA Amsterdam, The Netherlands; 2 Opris Dent SRL, Sibiu, Romania

**Keywords:** Neutrophils, Oral health, Apoptosis, Degranulation, Reactive oxygen species

## Abstract

**Purpose of Review:**

Oral health is maintained in a dynamic equilibrium between the host immunity and the oral microbiome. Oral polymorphonuclear neutrophils (oPMNs) are important innate immune cells in the oral cavity.

**Recent Findings:**

The oPMNs play a co-controlling part in the maintenance of oral equilibrium. In human saliva, the oPMNs integrity is preserved, and their function remains unaffected. In general, oPMNs are in a higher state of baseline activation compared to peripheral PMNs. However, in periodontitis, the oPMNs’ activation state can result in excessive release of damaging molecules in the extracellular environment.

**Summary:**

The presence of oPMNs may unwittingly negatively impact the integrity of the oral tissues. While most of the oPMN functions occur intracellularly, release of their potent active mediators into the extracellular environment may jeopardize oral homeostasis and its integrity. The dual nature of oPMNs, both beneficial and detrimental, remains a challenging and understudied topic.

## Introduction

The oral cavity holds a mucosal barrier, which is considered to be one of the main ecological habitats of the human body [1]. This oral mucosal barrier is under continuous microbial exposure as well as under repetitive mechanical force from chewing and hygiene habits, and is therefore distinct from the other human barrier sites.

In the case of any perturbation, the first line of defense in the continuous challenge between the host and microbes is our innate immune system [2]. In order to maintain equilibrium between the host and the microorganisms, an acute inflammatory response is initiated as a physiological response. This is characterized by the recruitment of innate immune cells within the host tissues at a challenged site. The interaction between various leukocytes and the microbiome has received high interest in the field of immunology. However, our understanding of the underlying cellular and molecular mechanisms still remains incomplete.

Within the blood circulation, polymorphonuclear neutrophils (PMNs) are the most abundant leukocytes (± 60%) and have evolved as specialized cells that are able to kill invading microorganisms [3]. Furthermore, PMNs are constantly present on all mucosal surfaces and constantly migrate into the oral cavity to maintain equilibrium [4•]. Paul Ehrlich discovered PMNs at the end of the nineteenth century [5]. After a series of cell divisions, the hematopoietic stem cells mature into neutrophilic cells, which eventually form the segmented PMNs with their characteristic multi-lobulated nuclei and enzyme-filled granules [3, 6]. PMNs are approximately 10–12 μm in size and are one of the first cells to arrive at a challenged (oral) site. To maintain a sufficient pool of circulating PMNs, a daily number of 1.0 × 10^11^ are continuously produced and are able to respond rapidly to pro-inflammatory signals, which they receive via cytokines, chemokines, Toll-like receptors, and Fc-receptors [3, 6].

Although the oral cavity is heavily colonized with microorganisms, overt infections rarely occur as long as an active equilibrium is maintained between the oral microbiota and host immune responses. With the PMN being the predominant leukocyte effector cells, evaluating their contribution in the healthy oral cavity can help in understanding their role in the pathological changes associated with oral conditions. Surprisingly, (oral) health is still often confused with the absence of (oral) disease. This has led to a body of literature focusing either on (I) PMNs from the blood circulation, or on (II) oral PMNs (oPMNs) in relation to inflammation or disease. This review emphasizes the role of oPMNs in physiologic, homeostatic oral conditions.

## What Is the Oral PMN? One Type, Or Many?

Since their discovery, PMNs have garnered broad interest and we have come to realize their important role in innate immune responses. Throughout the years, oPMNs have been isolated from several niches within the oral cavity. As an example PMNs in saliva [7], PMNs from oral/mucosal tissues [8••] and PMNs in crevicular fluid [9] have all been reported and interpreted as oPMNs. A clear definition for the oPMN seems to be lacking, leading to various and even contradicting characteristics that are being attributed to these cells. This makes the available knowledge on oPMNs more complex and sometimes even confusing. One could question if there is a need for a well-defined oPMN and what the biological repercussion would be. Presumably, a clear definition for oPMN subsets based on their source could lead to distinct oPMN phenotypes including distinct functional characteristics.

In contrast to other members of the innate immune system, the idea of PMN heterogeneity has received less attention. Classically, blood PMNs were considered to be a homogenous population, comprised of terminally differentiated cells with unique properties, such as a relatively short lifespan, absence of proliferative capacity, ROS production capabilities, and a limited capacity for cytokine release [3, 10]. These observations have sustained the traditional concept of PMN homogeneity. Over the last decade, more evidence has emerged regarding the phenotypic heterogeneity amongst PMNs from the peripheral circulation [11–13]. PMN subsets can be differentiated based on their phenotypic characteristics and in their capacity for antimicrobial activity. For example, priming and activation of PMNs is defined as a different state of cells that belong to the same phenotype. Another example was shown by Leliefeld and co-workers [13], which investigated intraphagosomal containment of live pathogens and showed that the phagocytic responses differed amongst the PMN subsets. An interesting review was recently published describing the current view on PMN heterogeneity with respect to both phenotype and function in health and disease [14••]. In addition to the knowledge gained on peripheral PMN subsets, the existence of oPMN subpopulations with distinct phenotypic and functional characteristics has also been reported previously [15•]. Since the oral cavity consists of a combination of healthy oral tissue sites and challenged oral tissue sites (e.g., local sites with inflammation), PMN heterogeneity therefore seems highly likely in the oral cavity.

In addition to the various activation states in tissue, oPMN subsets can also be different based on their migration route towards the oral cavity and therefore the age of the PMNs. The oPMNs consist of a combination of (I) blood PMNs that have emerged from the capillaries, for instance due to mechanical force or trauma, (II) migrated PMNs that emerged through the (ulcerated) pocket and junctional epithelium around the teeth (i.e., periodontal mucosa), (III) actively transmigrated PMNs that have undergone a longer and more complex migration route through mucosal tissues not associated with teeth, and (IV) migrated PMNs that have entered the oral cavity via other sources (for instance via sputum or tonsils). Although immunophenotyping of oPMNs is still an undiscovered area, heterogeneous cell populations are to be expected. However, it still remains unclear whether the different subset will also differ in their functional mechanisms and responses.

## Recruitment: Traveling from Blood to the Oral Cavity

PMNs can immediately be recruited to a challenged site to interact with invading pathogens [10, 16]. The release of PMNs from the bone marrow is tightly regulated [3]. After maturation in the bone marrow, PMNs enter the circulation and are able to migrate towards tissue upon activation. This process, called PMN recruitment, is a cascade mediated by the interaction of the PMN and the endothelium [3, 10]. While the majority of the PMNs will transmigrate paracellular using the endothelial cell-cell junctions, approximately 10% will migrate through the endothelial cell, in a process called transcellular migration [17]. Under normal physiological conditions, a pool of PMNs can be found within the intestines, the lungs, the liver, and the spleen [18–20]. The reason why PMNs are specifically concentrated within these locations is still unclear; however, it has been suggested that the presence of PMNs contributes to the equilibrium and immunological tolerance within these organs. Through constantly surveying and patrolling various tissue sites, PMNs may act as a monitor by seeking out for indications of microbial invasion or tissue damage [10, 21].

**Fig. 1:**
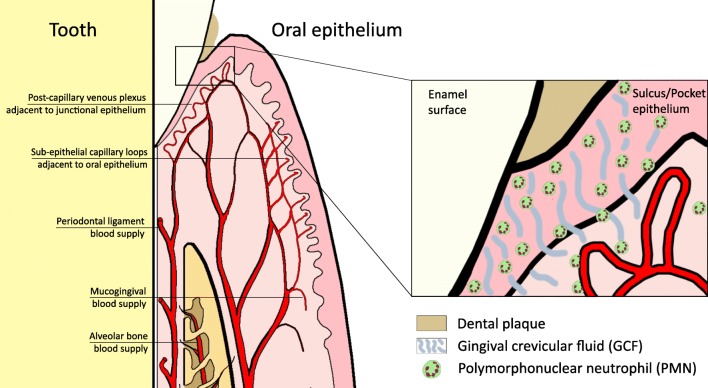
PMNs constantly extravasate from the circulation into the gingival sulcus/pocket. From here, PMNs can enter the oral cavity and mix with oral fluids

Comparable to these organs, oPMNs may have a similar function within the oral cavity [8••]. In the oral cavity, the periodontium is the tooth-supporting organ, which comprises of several anatomical structures surrounding the teeth. These structures include the gingiva, the periodontal ligament, the root cementum, and the alveolar bone. The periodontal ligament is the soft connective tissue interposed between the root of each tooth and the inner wall of the alveolar socket. From histological analysis of the periodontal tissues, it appears that the PMNs mainly leave the gingival blood supply surrounding the teeth, pass through the extravascular connective tissue, enter the junctional epithelium and pocket epithelium sulcular via the external basal lamina, and migrate preferentially along the central and tooth-related portions of the junctional epithelium until they reach the sulcus/pocket bottom [22]. Subsequently, PMNs mix with the fluid present in the gingival sulcus, also known as gingival crevicular fluid, which constantly flows into the oral cavity (Fig. 1).

Prior to the 1960s, the origin and the mechanism of PMN entry in the oral cavity were unknown. Since then, several studies have measured the migration rate of PMNs into the oral cavity through the gingival crevicular fluid [23–25]. The oPMN migration rate was shown to correlate with the level of local periodontal inflammation; however, the analysis was done on a tooth-by-tooth basis and not on a patient level. More recently, oPMN numbers have been reported on an individual level [4•, 15•, 26]. In addition, their numbers also seem to depend on the amount of teeth present, resulting in a decrease of oPMNs as subjects lose their teeth [27–30]. Throughout the years, many different research groups have focused on the isolation and quantification of oral PMNs and other leukocytes using various sampling techniques. The major observation of all of these studies is that all reported a consistent relative cell count, with the PMN being the predominant immune cell isolated from various oral fluids [4•, 15•, 26–36].

Interestingly, several studies also reported an increase of peripheral PMNs in patients with periodontitis [37–40]. The authors suggested the possibility of an increased availability of circulating PMNs that can actively migrate towards the inflamed periodontium. More recent reports, however, have observed the opposite showing varying oPMN numbers, while the number of circulating PMNs remained constant [4•, 30, 41]. We speculate that the majority of the oPMNs have entered the oral cavity by a process called diapedesis [42, 43]. Diapedesis is an active process that involves several migration steps out of the blood vessels from the circulatory system, followed by their movement through the junctional and sulcus/pocket epithelium surrounding the teeth. It was previously observed that the increasing pocket probing depths around the teeth with gingivitis or periodontitis correlates with PMN counts obtained from saliva [44]. PMN recruitment towards the oral cavity is highly dependent on the local (oral/gingival) conditions, where PMNs migrate towards a chemotactic gradient of chemokines and microbial chemoattractants, such as FMLP. We therefore suggest that the oPMN counts reflect the oral inflammatory conditions locally [8••, 26], while the numbers of PMNs in the blood circulation reflect the septic inflammatory status of an individual.

## Response: Duality in Promoting and Inhibiting Periodontal Inflammation

From a traditional point of view, PMNs were considered as simple foot soldiers of the innate immune system with a restricted set of inflammatory responses. The importance of PMN functioning is best reflected when the equilibrium is lost, leading to progressive inflammation throughout the whole body including the oral cavity. As an example, PMNs lacking NADPH oxidase activity are unable to provide a respiratory burst, resulting in the inability to kill microorganisms effectively [45, 46]. As a consequence, patients lacking the respiratory burst capability are profoundly immunodeficient and present with frequent acute and chronic infections.

In periodontitis, several studies have previously reported the presence of activated peripheral blood PMNs, which are possibly in a hyperactive state [47, 48]. An intrinsic increase of ROS production was therefore proposed for patients with chronic periodontitis as a susceptibility trait, showing excessive release of damaging molecules by the PMNs [47]. While most of these studies have used luminol-enhanced chemiluminescence in the past for the measurement of ROS generation (intra- and extracellular), nowadays flowcytometric analysis is becoming the preferred method of analysis, which is applicable in samples with low cell density, such as in our oral rinse samples, and is able to measure samples at a single cellular level. Recently, oPMN function was evaluated in periodontitis using flowcytometry [15•, 41]. The oPMNs in periodontitis patients display sufficient functionality as shown by their responsiveness after ex vivo stimulation. The question remains whether in periodontitis, the presence of increased oPMN numbers, and their excessive release of ROS and proteases can be detrimental to the oral mucosa integrity.

Periodontal homeostasis can be disrupted by a variety of host- or microbe-related factors. Historically, it was believed that periodontal inflammation was mainly driven by bacterial influx. The “Ecological Plaque hypothesis” that was proposed in 1994 suggested that an imbalance in the microflora allows for the overgrowth of disease-related microorganisms [49]. Recently, it has been demonstrated that the establishment of inflammation can induce changes in the composition of subgingival microbiota [50]. Moreover, chronic and hyperactive immune responses can additionally provide a rich and favorable ecological system where periodontitis-associated microorganisms can thrive. By affecting host response mechanisms, microorganisms can influence the stability and dynamics of the biofilm and induce a shift to a more pathogenic composition. Periodontal inflammation is therefore associated with microbial dysbiosis and a host inflammatory response to the microbial challenge, resulting in the degradation of the periodontium. Pathogenesis of periodontitis is characterized by an altered or aberrant host response to the presence of microorganisms, leading to immune cell-mediated self-destruction (mostly by PMNs) of the periodontal tissues [51]. Of note, not all patients will develop periodontitis and not all patients with periodontitis will result in total loss of their teeth. In the current understanding of periodontitis pathogenesis, differences in the severity and disease pattern can therefore be explained by susceptibility to periodontitis and a persistent inflammatory reaction due to multiple host- and microbe-related factors [51, 52].

PMNs are now acknowledged as multifaceted effectors, capable of a vast array of specialized functions, like phagocytosis (intracellular killing of microorganisms) and more recently NETosis [52]. Currently, the oPMNs have not been investigated for their NET formation capability. However, PMNs isolated from blood are able to form NETs in response to oral pathogens, from which certain microorganisms (e.g., *Veillonella parvula* and *Streptococcus gordonii*) stimulated higher ROS levels and NET formation compared to others [53]. Interestingly, influx of blood PMNs was also shown into dental plaque by Hirschfeld and co-workers [54], and also showed that oral bacteria were able to trigger intra-biofilm NET release and release of intracellular proteins [54, 55]. Formation of NETs may therefore also represent an additional functional mechanism for microbial trapping and killing by oPMNs in the oral cavity.

### Phagocytosis and Intracellular Killing

Several studies have recently reported on oPMNs’ functioning, and found that in vivo oPMNs retain most of their functional capacity within the healthy oral cavity [4•, 15•, 30, 41, 56]. PMNs are able to bind, ingest, and kill invading microorganisms through a process known as phagocytosis and are therefore considered as “professional phagocytes” [57]. Both peripheral PMNs and oPMNs are functionally able to execute phagocytosis; in the presence of saliva, oPMNs remain integral in their functional responses [58••]. Several receptors are present on the PMNs’ surface, which initiates the phagocytic signaling pathway. Phagocytosis is significantly enhanced by the presence of opsonins such as antibodies or complement factors on the microbial surface. Receptors specific for the Fc-region of antibodies include CD64 (FcγRI, IgG receptor), CD32 (FcγRIIa, low-affinity IgG receptor), CD16 (FcγRIIIb, low-affinity IgG receptor), and CD89 (FcαR, IgA receptor) [59, 60]. Furthermore, PMNs recognize complement-opsonized microorganisms using the complement receptors CD35 (CR1), CD11b/CD18 (CR3), and CD11c/CD18 (CR4) [61]. An opsonized microbe or particle binds to these surface receptors, which leads to its internalization into the PMN within a membrane-bound vacuole known as a phagosome.

The antimicrobial responses by PMNs have conventionally been distinguished as either oxygen-independent or oxygen-dependent [19, 45]. Both pathways have been identified in oPMNs, which are outlined below. In general, during internalization, PMNs mobilize their internal granule populations to merge with the phagosome [61, 62]. This oxygen-independent killing mechanism relies upon the microbicidal molecules situated within these granules. Oxygen-dependent killing is exerted through the production of reactive oxygen species (ROS) and are released in a process called the respiratory burst [61]. The joint composition of microbicidal agents and ROS will enrich the phagosome, creating a highly lethal intraphagosomal environment for the killing of ingested microorganisms.

### Oxygen-Independent Killing

PMNs possess a cocktail of antimicrobial agents. These include α-defensins, cathepsins, elastase, lysozyme, proteinase-3, and lactoferrin [61, 62]. It has been well established that these agents are located within distinct granule subsets of the PMN. The most common classification of granules is based on the presence of characteristic granule proteins: primary—azurophilic granules (identified by myeloperoxidase), secondary—specific granules (identified by lactoferrin), and tertiary—gelatinase granules (identified by gelatinase). An example of this classification with a list of identified constituents can be found in Table 1. Several studies recently investigated the oPMNs’ capacity for degranulation using various cluster of differentiation (CD) markers [4•, 15•, 41, 56, 64••]. By measuring various CD markers associated with active degranulation, the oPMNs were observed to be in a state of upregulated baseline expression, relative to peripheral PMNs. The studies on degranulation all reported that increased exocytosis occurs in the oPMNs’ granular contents. Recently, it was shown that lower levels of degranulation markers were observed during the initiation of gingivitis in an experimental gingivitis model [64••]. The lower levels of the observed CD markers suggest downregulation of the oPMNs’ activation state with minimal degranulation, suggesting the possibility of a more controlled oPMN response probably due to a sufficient number of oPMNs relative to the bacterial load present. In periodontitis, however, it was observed that various CD markers associated with oPMN activation is even more increased relative to the baseline expression levels seen in oral health [15•]. Interestingly, remnants of PMN membranes were also observed in supragingival dental plaque [54]. In addition, PMN-associated proteins such as myeloperoxidase (MPO), elastase (ELA-2), and cathelicidin LL-37 were detected in the vicinity of the PMN remnants, from which the first two are exclusively in PMNs. This suggests that PMNs are involved in host-biofilm control, although their specific contribution is still in need of further investigation. In early gingivitis, oPMNs may be able to contain the bacteria due to their increasing numbers without resorting to their normal full complement of functions, whereas in periodontitis the quantity and the quality of the biofilm might be overwhelming the oPMNs causing them to resort to the full extent of their resources, or triggering apoptosis, senescence, or degranulation.

**Table 1:** PMN granule contents stratified according to granule subsets and secretory vesicles

	Primary granules (Azurophil)	Secondary granules (Specific)	Tertiary granules (Gelatinase)	Secretory vesicles
Membrane-bound proteins	CD63	CD11b/CD18	CD11b/CD18	Alkaline phosphatase
	CD68	CD15	Cytochrome_*b558*_	CD10
	Presenilin 1	CD66	Diacylglycerol deacetylating enzyme	CD11b/CD18
	Stomatin	CD67	fMLP-R	CD13
	V-Type H^+^-ATPase	Cytochrome_*b558*_	Leukolysin	CD14
		fMLP-R	NRAMP-1	CD16
		G-protein_α_-subunit	SCAMP	CD45
		Laminin-R	SNAP-23, -25	CR1
		Leukolysin	uPA-R	C1q-R
		NB1 antigen	VAMP-2	Cytochrome_*b558*_
		19-kDa protein	V-Type H^+^-ATPase	fMLP-R
		155-kDa protein		Leukolysin
		Rap1, Rap2		VAMP-2
		SCAMP		V-Type H^+^-ATPase
		SNAP-23, -25		
		Stomatin		
		Thrombospondin-R		
		TNF-R		
		uPA-R		
		VAMP-2		
		Vitronectin-R		
Intra-granular matrix proteins	Acid β-glycerophosphatase	β_2_-Microglobulin	Acetyltransferase	Plasma proteins
	Acid mucopolysaccharide	Collagenase	β_2_-Microglobulin	
	α_1_-Antitrypsin	CRISP-3	CRISP-3	
	α-Mannosidase	Gelatinase	Gelatinase	
	Azurocidin	hCAP-18	Lysozyme	
	BPI	Histaminase		
	β-Glycerophosphatase	Heparanase		
	β-Glucuronidase	Lactoferrin		
	Cathepsins	Lysozyme		
	Defensins	NGAL		
	Elastase	uPA		
	Lysozyme	Sialidase		
	Myeloperoxidase			
	*N*-acetyl-β-glucosaminidase			
	Proteinase-3			
	Sialidase			
	Ubiquitin-protein			

*BPI* bactericidal permeability-increasing protein, *CD* cluster of differentiation, *CR* complement receptor, *CRISP* cysteine-rich secretory protein, *fMLP* formylmethionyl-leucyl-phenylalanine, *hCAP* human cathelicidin protein, *NGAL* neutrophil gelatinase-associated lipocalin, *nRAMP* natural resistance-associated macrophage protein, *R* receptor, *SCAMP* secretory carrier membrane protein, *SNAP* synaptosome-associated protein, *VAMP* vesicle-associated membrane protein, *TNF* tumor necrosis factor, *uPA* urokinase-type plasminogen activator (table adapted from Faurschou and Borregaard 2003) [63]

### Oxygen-Dependent Killing

Oxygen radicals and their reaction products are collectively called reactive oxygen species (ROS). PMNs can produce high levels of ROS as a consequence of NADPH oxidase activity, and can be released both intracellular and extracellular. The NADPH oxidase is a transport system that transfers electrons from NADPH to form superoxide and subsequently hydrogen peroxide and hydroxyl radicals. This process is referred to as the respiratory burst [65] and is an important functional mechanism that serves multiple purposes. Besides the destruction of microorganisms, the respiratory burst is known to be involved in the activation of PMN enzymes, chemotactic cell signaling, various forms of cell death, and modifications of cellular processes [66]. A wide array of responses ranging from undetectable levels to high levels of ROS can be produced by the PMNs in a very short period of time (within 30 s of cell activation) [67].

For oPMNs, it has been well established that they are also functionally capable of ROS production, both intracellular and extracellular as described above [4•, 12•, 45]. This might be important in the maintenance of oral homeostasis from several points of view. First, an excessive transmigration of PMNs towards the oral cavity can compromise the barrier function of the oral epithelia, leading not only to higher numbers, but allowing also for bacterial products to penetrate the tissues. In addition, the total amount of extracellular released products that originate from the PMN can compromise the epithelial surfaces further by causing damage during the passage through the oral tissues (I) and after migration when interacting with the epithelial surfaces (II) [68, 69]. A similar mechanism has been suggested for diseases such as inflammatory bowel syndrome and acute lung injury [70, 71]. This phenomenon may occur in a similar way within the oral cavity. An increase in oPMN numbers, antimicrobial molecules, and ROS may therefore have consequences for the integrity of the oral mucosa. It has additionally been suggested for oPMNs that these cells possibly represent a distinct subset from the peripheral PMNs, which acquire phenotypic traits during the migration process towards the oral cavity [15•, 41]. In this way, oPMNs can partly be responsible for maintaining the chronicity of gingivitis and periodontitis and the tissue damage for the latter condition, both due to their increased presence and the release of damaging molecules into the extracellular environment.

## Resolution: Beneficial Suicide*?*

Over 10^11^ circulating PMNs undergo programmed cell death each day, in a process called spontaneous or constitutive apoptosis [72]. In humans, this clearing mechanism maintains the homeostatic level of functional PMNs and is considered paramount to the resolution of acute inflammation, and minimizing potential collateral tissue damage. The bone marrow can act as a PMN reservoir, with a stored pool up to 10 times the number of circulating PMNs that can rapidly be released. Interestingly, it is suggested that PMNs circulate for only 4–12 h after their release from the bone marrow [18, 73]. Although the reason for this short lifespan is still unclear, this may ensure the cells integrity and makes the PMN amongst the shortest-living cells in the human body. PMNs spontaneously undergo apoptosis in vivo, after their migration from the vascular compartment to the tissue.

Apoptosis (programmed cell death) has been suggested as a mechanism for the internal regulation of PMN homeostasis. In order to maintain a steady PMN level, spontaneous apoptosis occurs in the absence of activation, which is described for PMNs in the circulation [74]. In addition, apoptosis is associated with an overall diminished functional capacity, like impaired chemotaxis, phagocytosis, degranulation, and ROS production [75, 76]. The PMNs’ functioning may therefore depend on a balance between anti- and pro-apoptotic signals. Upon migration to a challenged site or an inflammatory focus, the PMNs’ longevity can increase upon activation as a result of cytokines, growth factors, and bacterial products [18, 77]. This extended lifespan is also highly likely occurring for the oPMNs. A longer lifespan may allow for the oPMNs to carry out more complex activities in the oral cavity and on the mucosal surfaces, and thereby contributing to the oral equilibrium by preventing microbial invasion, with the risk of their persistent presence leading to collateral tissue injury. Since the activated PMN may also hamper the mucosal integrity, timely removal can prevent unwillingly damage of host tissues caused by the inappropriate or excessive presence of activated PMNs. In addition, clearance of apoptotic PMNs can restrict the extent of possible tissue damage by actively suppressing PMNs’ pro-inflammatory cytokines secretion, such as tumor necrosis factor (TNF)-α, and drive production of the anti-inflammatory cytokines, such as interleukin (IL)-10 and transforming growth factor (TGF)-β by pro-resolving macrophages [78]. Clearance of oPMNs by for example swallowing and shedding of the gingival epithelium can therefore be beneficial by preventing the release of highly toxic PMN components into the surrounding tissue, and also by promoting tissue healing and suppressing the inflammatory response. Whether the exact persistence time of the oPMNs is really longer compared to the longevity of the PMNs from the blood circulation still remains to be seen. Direct observation of real-time PMN trafficking within the mucosal vasculature may help to clarify this issue [79].

## The Oral Barrier: Are oPMNs a Double-edged Sword for the Oral Tissues?

The interaction between PMN and the microbiome at various human anatomical sites has received high interest in the field of immunology. Nowadays, increasing evidence supports the notion that mucosal commensals can affect the presence of PMNs [80, 81]. While our understanding of the underlying cellular and molecular mechanisms still remains incomplete, it has been suggested that microorganisms can influence the PMNs numbers by activating their pattern recognition receptors. In this way, a steady-state PMN recruitment is promoted at the healthy oral barrier [8••].

In the mouth, the barriers to prevent microbial invasion are the oral mucosal surfaces that cover the jaws, the cheeks, the tongue etc. The oral mucosa is considered to be one of the main ecological habitats of the human body [82]. While some oral sites are particularly thin and highly vascularized (like the sublingual area), other areas possess a thicker layer of keratin protection that are subjected to mechanical stimulation (like chewing). These areas are packed with oral microorganisms, which have been shown to form biofilms with a complex structural organization [83]. A particular vulnerable site in the oral cavity is the epithelium of the gingival crevices surrounding the teeth. While this location may function as a port of exit for the oPMNs to enter the oral cavity, microorganisms can also use the same site as a port of entry. This site is therefore under continuous microbial exposure, which needs to be critically guarded. It seems only logical that PMNs are needed to operate at the gingival barrier in health (Fig. 1). Since these sites are additionally under continuous stress due to oral functions like speech, repetitive mechanical force from chewing, and hygiene habits, the oral cavity and the oral mucosal barrier are therefore distinct from other barrier sites in the human body.

The delicate balance in the mouth between the oral microbiome and the innate immunity, including an important role for oPMNs, is best reflected when equilibrium is lost, leading to progressive inflammation. As an example, PMNs lacking NADPH oxidase activity are unable to provide a respiratory burst, resulting in the inability to kill microorganisms effectively [13, 46]. As a consequence, patients lacking the respiratory burst capability are profoundly immunodeficient and present with frequent acute and chronic oral infections. On the other hand, similar to inflammatory bowel disease, the persisting presence of oPMNs on the mucosal surface of the periodontal lesion may also have implications for the continuation and progression of periodontitis [41, 63, 84, 85]. Despite our increasing understanding regarding host-microbiome co-existence, the underlying mechanisms to maintain equilibrium are not well understood. Thus, more research is needed to further understand the interrelations between oPMNs and microbiota in the healthy oral ecosystem, and to assess the changes occurring in the ecosystem when challenged and facing dysbiosis.

## Conclusions

Many factors can influence the course, pattern, and duration of the innate immune responses, of which the PMNs form an important aspect. Even though it seems likely that oPMNs play a co-controlling part in the maintenance of oral equilibrium, an inappropriate or excessive infiltration of activated PMNs can result in excessive release of antimicrobial proteins and ROS production. The oPMNs are preserved in saliva and saliva does not seem to compromise the PMN integrity and their functionality. In addition, oPMNs are in a higher state of baseline activation compared to peripheral PMNs. In periodontitis, oPMNs may even transcend to a highly activated state possibly resulting in a hyperactive state. While most of the destruction of phagocytosed particles and microbes still occur intracellularly, it is hypothesized that the extensive release of damaging molecules into the extracellular environment can unwillingly result into collateral damage of the hosts’ tissues. Prolonging the lifespan of the oPMN seems to ensure the presence of primed PMNs at the oral sites, while timely removal is considered essential for the maintenance of cellular and tissue homeostasis. Thus, only the synergy of the hosts’ defense arsenal can provide a peaceful co-existence, i.e., balance, of the host with its oral environment.
